# Geometric phase coded metasurface: from polarization dependent directive electromagnetic wave scattering to diffusion-like scattering

**DOI:** 10.1038/srep35968

**Published:** 2016-10-24

**Authors:** Ke Chen, Yijun Feng, Zhongjie Yang, Li Cui, Junming Zhao, Bo Zhu, Tian Jiang

**Affiliations:** 1 Department of Electronic Engineering, School of Electronic Science and Engineering, Nanjing University, Nanjing, 210093, China

## Abstract

Ultrathin metasurface compromising various sub-wavelength meta-particles offers promising advantages in controlling electromagnetic wave by spatially manipulating the wavefront characteristics across the interface. The recently proposed digital coding metasurface could even simplify the design and optimization procedures due to the digitalization of the meta-particle geometry. However, current attempts to implement the digital metasurface still utilize several structural meta-particles to obtain certain electromagnetic responses, and requiring time-consuming optimization especially in multi-bits coding designs. In this regard, we present herein utilizing geometric phase based *single* structured meta-particle with various orientations to achieve either 1-bit or multi-bits digital metasurface. Particular electromagnetic wave scattering patterns dependent on the incident polarizations can be tailored by the encoded metasurfaces with regular sequences. On the contrast, polarization insensitive diffusion-like scattering can also been successfully achieved by digital metasurface encoded with randomly distributed coding sequences leading to substantial suppression of backward scattering in a broadband microwave frequency. The proposed digital metasurfaces provide simple designs and reveal new opportunities for controlling electromagnetic wave scattering with or without polarization dependence.

Recently, the ability to manipulate the electromagnetic (EM) waves has been significantly improved with the help of the emerging concept of metasurface. Unlike the bulk metamaterial, metasurface comprising a monolayer of engineered meta-particles can provide phase discontinuities across the surface thus both the reflected and the refracted EM wavefront can be completely manipulated at will. By spatially varying the characteristics along the surface, one can elaborately engineer metasurfaces with desirable features, and a variety of devices with peculiar functionality have been demonstrated, including invisibility skin cloak^1^,^2^,^3^, ultrathin flat lens^4^,^5^,^6^,^7^,^8^,^9^, holography^10^,^11^,^12^,^13^, etc.

The geometric phase, or called the Pancharatnam-Berry phase^14^,^15^, is a quite promising approach for manipulating the phase behaviors of the meta-particles. Compared to those designed by various forms of V-shaped^7^,^16^,^17^, C-shaped^18^ or more sophisticated constructions^19^,^20^ with varying structural parameters according to the diverse spectral responses, the meta-particles operated with geometric phase can allow a full manipulation of 360° phase shift only by rotating the same meta-particle with certain geometric orientation. Particularly, if the meta-particle has an local orientation angle denoted by *α*, a circularly polarized EM incident wave will flip its helicity in transmitted operation while preserve it in reflected operation, and imparted with an abrupt geometric phase change of ±2*α* 4,5,14,15,21,22,23,24,25,26,27,28,29,30. Apparently, such geometric phase provides an additional degree of freedom in the gradient metasurface design associated with polarization-dependent EM wave manipulation including photonic spin Hall effect^25^, beam split^25^,^26^, as well as other functional devices like helicity-selective holograms^27^,^28^,^29^, vortex beam generator^30^, etc.

More recently, by selecting two particular meta-particles with out of phase reflection properties as the digital bits of “0”, and “1”, and arranging their distribution in the metasurface with optimized coding sequences, people are able to create the so-called digital or coding metasurface to achieve anomalous EM wave reflection and scattering^19^,^31^,^32^,^33^,^34^,^35^. Although the digital or coding metasurface has simplified the design and optimization procedures due to the digitalization of the meta-particle geometry, current attempts to implement the digital metasurface still involve several meta-particles with different geometrical structures therefore require time-consuming optimization especially in multi-bits coding designs where the computation complexity increases exponentially with the bit number^19^.

Polarization is one of the basic properties of EM wave, and polarization dependent control could provide more degrees of freedom in many practical applications. Here in this paper, we try to employ the geometric phase concept to the digital metasurface design, which only contains a single structured meta-particle to achieve either 1-bit or multi-bits coding elements that has distinct scattering behaviors for different circularly polarized EM waves. The employed meta-particle is based on a corrugated metallic meander line structure on top of a grounded dielectric substrate, which has highly anisotropy within a wide frequency band and the different digital bits are defined by rotating such meta-particle with certain angle. When applying regular coding sequences, the multi-bit digital metasurface can generate distinct directive scattering field highly dependent on incident polarizations. On the other hand, when encoded with randomized digital sequences the metasurfaces become polarization-insensitive and result in a diffusion-like scattering pattern with wideband low specular reflection, indicating its potential use in backward scattering reduction in stealth technique. Experiments are carried out on fabricated prototypes to validate the design principle as well as the simulated predictions, which show relatively good coincidence.

## Results

### Geometric phase coded digital metasurface

**Figure 1:**
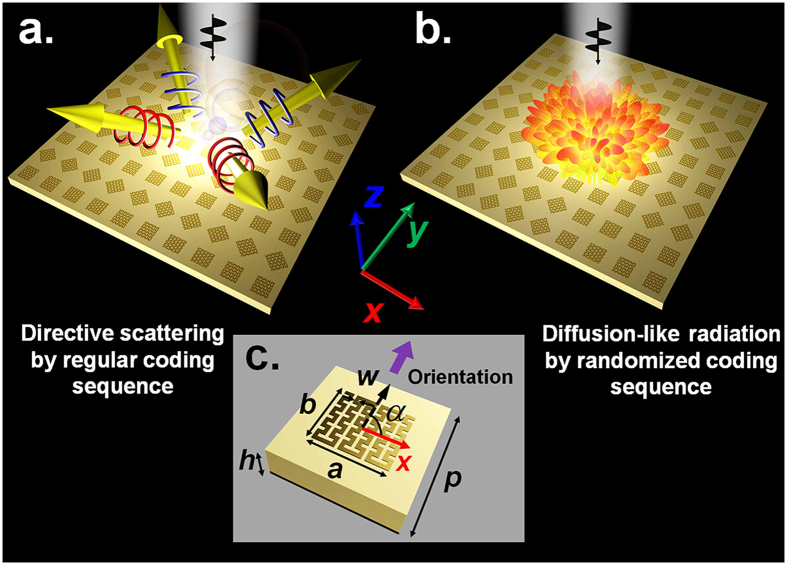
The geometric phase coded digital metasurface. (**a**) Distinct directive EM wave scattering dependent on the incident polarization by metasurface with regular coding sequence. (**b**) Polarization insensitive diffusion-like reflection by metasurface with randomized coding sequence. (**c**) Details of the corrugated meander line structured unit cell which is oriented at the rotation angle of *α* = 90° with respect to *x*-axis. The violet arrow represents the orientation of top metallic pattern. The optimized geometric configuration of the unit cell is by, in millimeter, *p* = 8, *h* = 2, *a* = 4.6, *b* = 4.25, and *w* = 0.25.

**Figure 2:**
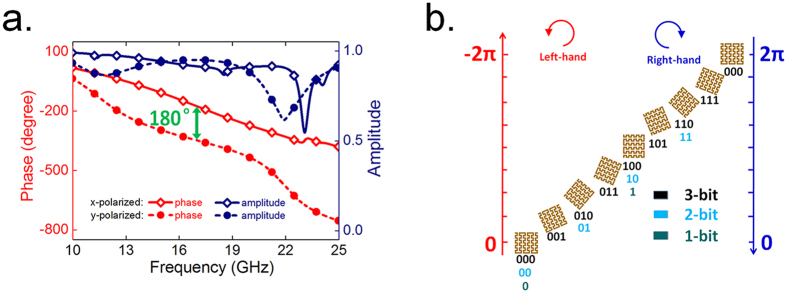
Performances of basic meta-particle and the definition of geometric phase coded bits. (**a**) Simulated reflection spectra of the unit cell shown in Fig. 1c under the illumination of linearly *x*- and *y*-polarized EM waves, respectively. (**b**) The definition of the bit elements of 1-bit, 2-bit, and 3-bit digital metasurfaces and their corresponding PB phase.

The metal inclusions composing the metasurface often act as strong scatters of EM wave and response extremely sensitive to their geometric patterns. By designing meta-particles with various metallic patterns, a wide range of spectral property can be accessed, as well as controlled anisotropy operating distinctly for different polarized EM waves. In our scheme, an elaborately designed metallic corrugated meander line pattern on top of a grounded dielectric substrate is used as the basic meta-particle. As schematically illustrated in Fig. 1, such meta-particle is anisotropy in the *x-y* plane therefore may provide polarization dependent response. Copper thin film with thickness of 0.018 mm is used to form the top metallic pattern and the ground plane, while the dielectric layer has a thickness of 2 mm with a relative permittivity of 4.3 and loss tangent of 0.025. We first analysis the EM response of the meta-particle structure. By optimizing the geometric parameters of the meta-particle, a nearly constant 180 degree reflection phase difference within a wide frequency band from 12 GHz to 21.5 GHz can be achieved between linearly *x*- and *y*-polarized incidences. Meanwhile, as shown in Fig. 2a, the EM wave reflection amplitude is kept near unity as there is little energy dissipation during the EM wave coupling and reflection. These results also indicate that the meta-particle can form a good half-wavelength plate operating in reflection mode. When illuminated by circularly polarized waves this kind of anisotropic structure can produce the so-called geometric phase shift, a phase shifts of *φ* = 2*σα* depending on the rotation angle *α* of its orientation, where *σ* = ± 1 is polarization sign corresponding to right-circularly polarized (RCP) or left-circularly polarized (LCP) incidences, respectively. This type of additional phase shift can be well interpreted by Pancharatnam−Berry (PB) phase^14^,^15^, which is recently employed in metasurface designs for developing various polarization dependent devices. This phenomenon provides an explanation of the 180 degree reflection phase difference for the two orthogonal polarized incidences in Fig. 2a. Since the reflection for circular polarized wave is delayed or advanced in phase by 2*α* due to the rotation of the meta-particle by an angel of *α*, the phase responses of a designed structure can be tuned to cover the full phase range of 360° only by rotation of the meta-particle.

In a digital reflective metasurface, several elements with different phase properties are utilized to form certain custom-tailored coding sequences to control the EM wave scattering. Practically, for a 2-bits case, the entire reflection phase coverage of 360° should be discretized into four levels, namely 0°, ±90°, ±180° and ±270°, to mimic the digital bits of “00”, “01”, “10”, “11”, respectively. For 1-bit and 3-bits cases, two or eight elements should be defined. Although there may be other definitions of digital metasurface, this procedure has been proved to be simple and efficient to control, and the recent digital metasurface designs are exclusively defined by this method^19^,^31^,^32^,^33^,^34^. Up to now, an *N*-bits digital metasurfaces usually require 2^*N*^ different meta-particles with optimized geometrics to achieve diverse phase shifts for certain incident wave. Here, we present a design scheme of digital metasurface utilizing geometric phase concept with a single anisotropic meta-particle of a corrugated meander line pattern illustrated in Fig. 1c. Here each bit element is represented by the same meta-particle with certain rotation angle. This kind of geometric phase digital metasurface would ease the design and optimization processes as well as offers more degree of freedoms to manipulate the wave reflections by their polarization-dependent phase responses.

In the definition procedure, the orientation angle *α* gradually increases from 0° to 180° with a fixed step-size as illustrated in Fig. 2b. For 1-bit, 2-bits or 3-bits metasurface the step-size of rotation angle for each constitution meta-particles is chosen as 90°, 45° or 22.5°, respectively. Since each digital bit has an opposite phase responses equaling to twice the local rotation angle for RCP or LCP EM waves, the digital meta-particles can be utilized to enable many polarization-dependent functionalities. Figure 1a schematically illustrates that regular coding sequences can result in incident polarization dependent directive scattering, while Fig. 1b indicates that the polarization insensitive diffusion-like reflections can be achieved by metasurface with randomized coding sequences. In the following, we will focus on the design of these two types of digital metasurfaces composed of the proposed geometric phase coded digital metasurface.

### Polarization dependent directive scattering by regular coding metasurface

**Figure 3:**
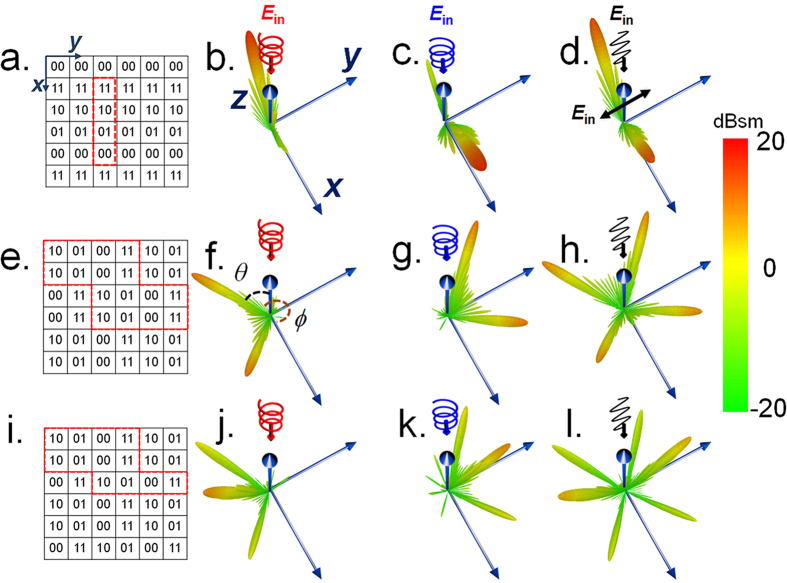
The 3D far-field radiation pattern of the digital metasurface at 17 GHz with regular coding sequences. (**a**) The coding sequence with a periodic reads 11, 10, 01, 00 is applied along *x*-direction while invariant along *y*-direction, and the corresponding scattering patterns in response to LCP, RCP, and linearly *y*-polarized EM waves are shown in (**b–d**), respectively. (**e**) The coding sequence with a periodic read 11, 10, 01, 00 is applied along *y*-direction, while with a phase difference of π along *x*-direction, and the corresponding scattering patterns in response to LCP, RCP, and linearly *y*-polarized EM waves are shown in (**f–h**), respectively. (**i**) The coding sequence with a periodic read 11, 10, 01, 00 is applied along *y-*direction, while a periodic of 3*p* along *x-*direction, and the corresponding scattering patterns in response to LCP, RCP, and linearly *y*-polarized EM waves are shown in (**j–l**), respectively. The coding bits circled by red dotted lines represent a repetition period along *x*- or *y*-direction.

It is commonly known that the wavefront as well as the field radiation pattern is the collective response of each individual elements of the metasurface, and arbitrary digital metasurface has a unique scattering field pattern according to its coding sequence and vice versa. Therefore, encoding the metasurface with pre-designed sequences, it may create the scattering field pattern as desired. Here, we start with 2-bits case to show the polarization-dependent properties in the EM field scattering manipulation by the proposed geometric phase coded metasurface. The employed metasurface consists of 25 × 25 = 625 elements with coding sequence of 11, 10, 01, 00, 11, 10, … along *x*-direction while invariant along *y*-direction. When it is exposed to a normal plane wave incidence, the resulted three-dimensional (3D) far-field radiation patterns are displayed in Fig. 3. The full wave simulation shows that a main lobe clearly appears at the elevation angle of −33.5° deviated from *z*-axis under the illumination of LCP incidence, but at an elevation angle of 33.5° for the RCP incident wave. This is mainly due to the spatially varying phase distribution on the metasurface having exactly opposite constant gradient between LCP and RCP waves, thereby leading to the far-field scattering EM wave deflected to two mirror-symmetric directions. The anomalous reflection angle (described by the elevation angle *θ* and azimuth angle *ϕ* in spherical coordinate as illustrated in Fig. 3f) of the directive scattering wave with respect to the surface normal (+*z* direction) can be precisely designed by the generalized Snell’s laws given by *θ* = |arcsin(*λ*ΔΦ/2π*p*)| = 33.5° and *ϕ* = 0° or 180°, where *λ* is the wavelength in free space at 17 GHz, *p* and ΔΦ represent the lateral size of unit cell (indicated in Fig. 1c) and the phase gradient (equals to ±π/2) according to the incident helicity^16^. Since a linearly polarized wave can be represented by the sum of two circularly polarized waves with opposite helicity, therefore, such digital metasurface can also be utilized to separate a linearly polarized incidence into two anomalously reflected circularly polarized beams, as illustrated in Fig. 3d.

The control over the scattering property by the digital metasurface is highly dependent on the applied coding sequences as well as the incident polarization. As other examples to demonstrate the capability of such digital metasurface in controlling the wave scattering, we present that the digital metasurface can be designed to generate distinct multiple directive beams according to the incident polarization. As illustrated in Fig. 3e, the metasurface has a constant phase gradient along *y*-direction while a phase difference of π with a period of two elements along *x*-direction. In this case, the scattering field will experience destructive cancellation in *y*-direction and be separated into two mirror-symmetrically orientated beams apart from the negative *y*-axis with angles of ±arcsin(*λ*/4*p*) = ±33.7° (corresponding to *ϕ* = 236.3° and 303.7°) for the LCP incidence according to the phased-array antenna theory, while the constant phase gradient along *y*-direction will deflect the scattering wave into the plane towards the negative *y*-direction with an angle of 33.5° deviated from *x-z* plane as shown in Fig. 3f. For the RCP incidence, the twin beams will shift to their mirror-symmetric directions with respect to *x-z* plane as shown in Fig. 3g. The coding sequence shown in Fig. 3i with a period of three elements along *x*-direction is utilized to generate three pencil-like beams orientated with angles of 0°, arcsin(*λ*/3*p*) = ±45.1° with respect to the *y*-z plane (corresponding to *ϕ* = 224.9°, 270° and 315.1°, respectively) for the RCP incidence. Similarly the constant phase gradient along *y*-direction acts as the polarization dependent factor to deflect the directive scattering wave to distinct directions dependent on the helicity of the incidence. As shown in Fig. 3j,k, for the RCP incidence, these three beams are deflected into the plane towards *y*-direction with an angle of 33.5° apart from *x-z* plane, while the beams will shift to their mirror-symmetric directions with respect to *x-z* plane under the illumination of LCP incidence. Besides, these two digital metasurfaces can generate four or six beams under the illumination of linearly polarized waves, as shown in Fig. 3h,i, respectively. Moreover, these metasurfaces are insensitive to the azimuthal angle of linearly polarization due to that the arbitrary linearly polarized wave can be divided into two circularly polarized waves with opposite helicity. The simulation results show in Fig. 3 agree with the theoretical analysis, which reveal that the metasurface with geometric phase coded elements could achieve distinct directive scattering waves highly dependent on the incident polarizations. We could expect that more diverse polarization dependent EM wave scattering can be envisaged by applying elaborately pre-designed coding sequences.

### Diffusion-like scattering by randomized coding metasurface

Benefitting from the geometric phase, we have successfully demonstrate the polarization dependent directive scattering of EM wave by metasurface with regular coding sequence. In the following, we will present that by appropriately encoding with randomly distributed bits, the metasurface slab can be easily tuned into a polarization-insensitive one with diffusion-like scattering property, which could significantly suppressing the backward scattering in a wide frequency band and may be potentially useful in stealth technique.

Unlike the specular reflection occurred at a planar interface between two isotropic common media, the diffuse reflection induced by the wavelength-comparable roughness at an interface will redirect the incidence into various directions. Apparently, the ultrathin planar version of such surface with diffusion-like reflection can be easily imitated by the proposed digital metasurface, since the phase responses across the interface can be controlled to destroy the specular reflection wavefront. In contrast to the pencil-like beams or diverse regular beams, the reflective metasurface considered herein is utilized to mimic the diffuse reflection to generate scattering to the whole upper half-space. The phase distribution on the metasurface therefore should be as random as possible to uniformly redirect the scattered waves into all directions so as to suppress the specular scattering.

**Figure 4:**
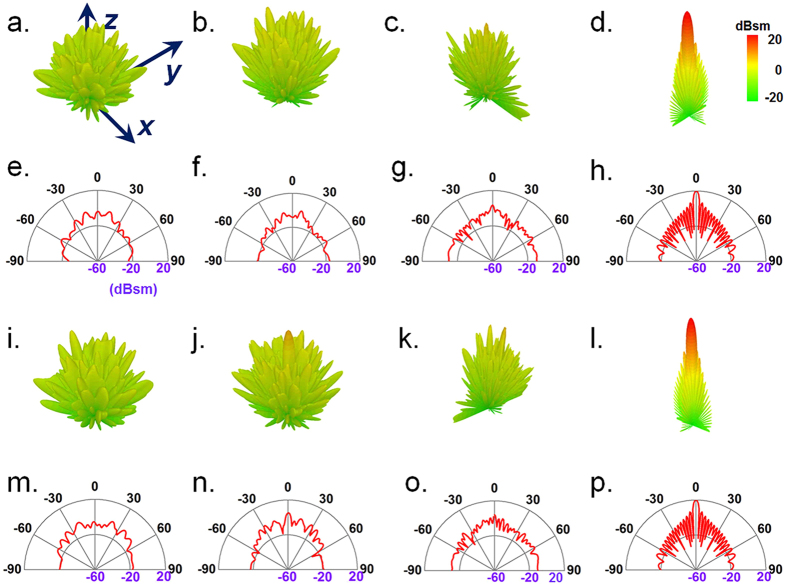
Simulated performances of the metasurface with randomized 2-bit coding sequences. The 3D scattering patterns under normal incidence of *x*-polarized EM wave for (**a–c**) the digital metasurface at 13.5 GHz, 17.5 GHz, and 21.5 GHz, respectively, as well as (**d**) the same-sized metallic slab at 21.5 GHz. The corresponding 2D counterparts in ***E***-plane are displayed in (**e–h**), respectively. The 3D scattering patterns under normal incidence of *y*-polarized EM wave for (**i–k**) the digital metasurface at 13.5 GHz, 17.5 GHz, and 21.5 GHz, respectively, as well as (**l**) the same-sized metallic slab at 21.5 GHz. The corresponding 2D counterparts in ***E***-plane are displayed in (**m–p**), respectively.

To realize a reflective metasurface with diffusion-like backward scattering, we use a super unit cell containing 2 × 2 same digital meta-particles, and uniformly extend them in a finite sheet of 200 × 200 mm^2^ according to a computer-generated pseudorandom coding sequence (schematically shown in Fig. 1b). Considering there are countless possibilities in determining the randomized coding sequences, here we only investigate a certain case to validate the diffusion-like scattering by the digital metasurface. The simulated results of 3D far-field radiation patterns under the normal illumination of a plane wave are presented in Fig. 4. It obviously shows that the EM wave scattered from an encoded metasurface are randomly distributed in the whole upper half-space, which is intrinsically different from that of a bare same-size-controlled metallic slab shown in Fig. 4d,i, where only a dominating specular reflection with low side-lobes is observed. The diffuse metasurface has a broad operation frequency bandwidth as the diffusion behavior can be clearly observed at 13.5, 17.5 and 21.5 GHz under linearly *x*- or *y*-polarized illuminations as shown in Fig. 4a-c or i-k, respectively. Actually, the backward scattering is greatly suppressed with at least 10 dB reduction along the surface normal, which means that less than 10% power gets bounced back across the entire frequency band ranging from 13.5 to 21.5 GHz. We also curve the radiation pattern in ***E***-plane at the above three frequencies to give a detailed comparison with that of bare metallic slab, as illustrated in Fig. 4e–h,m–p. Attributed to the disordered distribution of phase responses at the interface, enormous beams with significantly restrained energy are randomly scattered in various directions to the upper half-space.

The radiation from a metallic slab shows a pencil-like beam shaped scattering pattern owing to the in-phase responses at the surface which further result in constructive interference along the backward direction in far-field region. However, by replacing the metallic slab with randomly distributed digital elements to destroy uniform phase responses at the interface, the scattering waves will undergo complex interferences in a random manner, thus be redistributed to all directions similar to a light impinging onto a rugged surface. Hence, the radiation pattern should tend to be an omnidirectional scattering. However, the diffuse reflection in the presented scheme is not an ideal one as the radiation patterns shown in Fig. 4 are not uniformly distributed with same intention in all directions. This is mainly due to the finite size of the metasurface and the coding sequence which is not a real randomized one in practical design. Nevertheless, it has undoubtedly demonstrated the concept of mimicking a reflective rugged surface to redirect the incidences into various directions, as well as a substantial specular reflection reduction.

### Experiments verification

**Figure 5:**
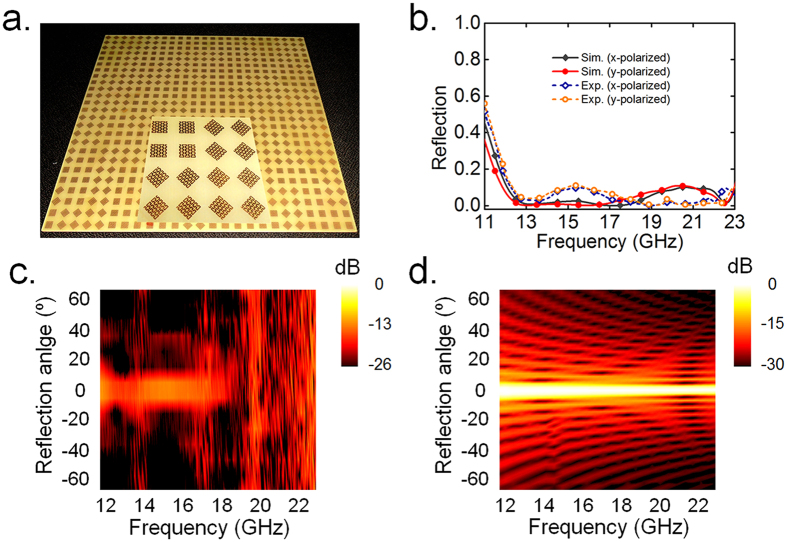
Fabricated sample of digital metasurface and the results of backward scattering. (**a**) The photograph of a 2-bit digital metasurface with randomized coding sequences. Inset shows the zoomed-view of fragments. (**b**) Simulated and measured frequency-dependent reflections of 2-bit digital metasurface under the normal plane wave illumination. (**c**) Measured broadband scattering pattern of the 2-bit digital metasurface with randomized coding sequences. (**d**) Simulated broadband far-field scattering of a bare metallic slab.

To validate the above concept and predictions, we fabricate a prototype of diffuse metasurface slab consists of digital elements with the aforementioned randomized coding sequence based on standard print circuit board (PCB) technique, as shown in Fig. 5a. The measurements are carried out in a standard microwave chamber and the reflection is calibrated to a same-dimensioned copper slab. As shown in Fig. 5b, the measured low specular reflection below 0.1 can be preserved from 12 to 23 GHz, which roughly coincides with that of full wave simulations both in the cases of *x*- and *y*- polarized incidences. The reduction in the band of 12–21 GHz is mainly caused by diffusion nature attributed to the spatially random phase distribution, while the reduction around 22 GHz is the collective results of the absorption and phase destructive cancellation since the EM resonances here in the meander line structure leads to energy dissipation either under *x*- or *y*-polarized excitations. To further describe the diffuse characteristics of the reflective metasurface, broadband radiation patterns in ***E***-plane are also depicted by detecting the scattering energy at various angles from −65° to +65° with an increment of 10° under the incidence fixed at the angle of −5°, as illustrated in Fig. 5c. Compared to the result of a bare metallic slab shown in Fig. 5d, where dominating specular reflection always appears at the angle of 5° across the entire band, clearly the scattering power from diffusion metasurface spread to various directions without strong energy in any particular direction, indicating a good diffuse scattering as well as a substantial specular reflection reduction.

**Figure 6:**
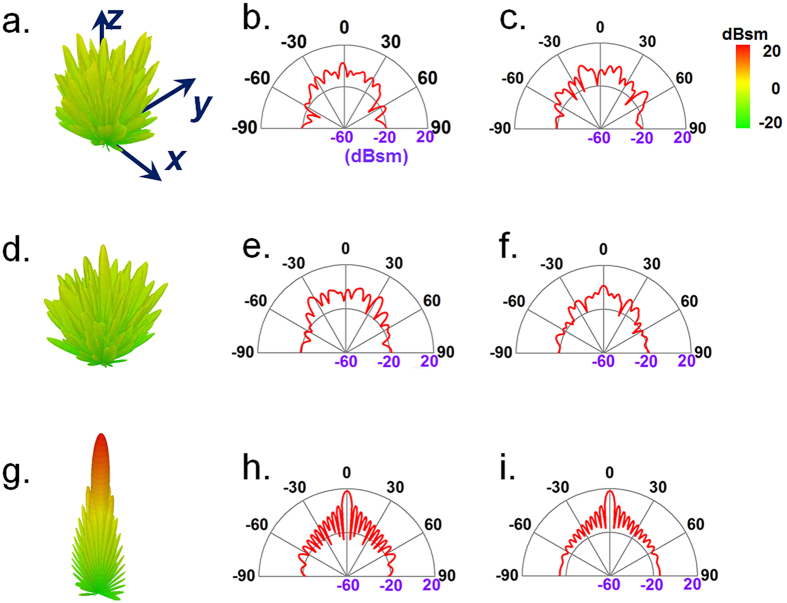
Simulated far-field scattering of 1-bit and 3-bit digital metasurfaces with randomized coding sequences. The scattering pattern of (**a–c**) 1-bit, and (**d–f**) 3-bit digital diffusion metasurface, as well as (**g–i**) bare metallic slab at 17 GHz. The left, middle, and right columns illustrate the 3D scattering patterns, their corresponding 2D patterns in ***E***-plane, and in ***H***-plane, respectively.

**Figure 7:**
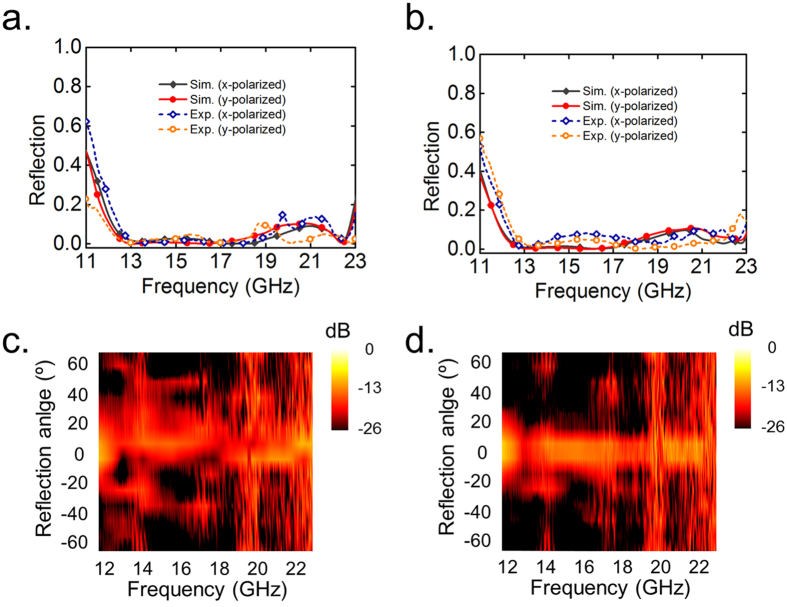
The simulated and measured results of 1-bit and 3-bits digital metasurfaces in wide frequency band. The simulated and measured frequency-dependent reflections of (**a**) 1-bit, (**b**) 3-bit diffusion metasurface under normal illumination. The measured far-field scattering pattern in ***E***-plane of the (**c**) 1-bit, or (**d**) 3-bit digital diffuse metasurface.

The digital metasurface as well as the diffusion behavior can be reproduced with 1-bit or 3-bits coding cases. We would emphasize that there is no extra calculation and optimization process necessary for multi-bits design, since the digital meta-particles are solely dependent on the orientation of corrugated meander line pattern. Based on the definition shown in Fig. 2b, we fabricate the metasurface slab with computer-generated randomized 1-bit or 3-bits coding sequence. The simulated 3D scattering patterns with their 2D counterparts in ***E***-plane and ***H***-plane under the illumination of a planar incidence at 17 GHz are illustrated in Fig. 6. Compared to that of a bare metallic slab which has pencil-like radiation pattern, good diffuse EM scattering in far-field region are obtained due to the randomized phase responses on the metasurface. To confirm the diffuse reflection by these digital metasurface, we have also experimentally measured the frequency-dependent reflections as displayed and compared in Fig. 7a,b, which roughly agree with the simulated predictions. The measured far-field radiation features show a broadband scattering energy reduction of more than 10 dB in all directions in the entire band from 12 GHz to 23 GHz. These results once again validate that the digital metasurface, either with 1-bit or multi-bits coding elements, is a simple but powerful approach to achieve broadband polarization-insensitive low backward scattering. Further optimization and flexible control of the working bandwidth could be realized by the smart methods proposed in ref. 36.

## Discussion

In summary, we present a new strategy to achieve either 1-bit or multi-bits geometric phase coded metasurface utilizing a single-sized corrugated meander line structure with different orientation angles. The design of multi-bits digital metasurface is significantly simplified since there is no extra optimization and calculation processes in the transitions from 1-bit to multi-bits. By encoding with pre-designed regular coding sequences, the metasurface produces distinct directive scattering patterns highly dependent on incident polarizations. While encoding the metasurface with randomized coding sequences to analogue rugged surfaces, polarization-insensitive diffusion-like reflections can be achieved over a wide frequency band. Experiments are carried out to confirm the diffuse metasurface, which agree well with the numerical predictions. The work presented herein provides a glimpse of the opportunities available for advanced EM functional devices based on geometric phase encoded digital metasurface, which may find particle applications in the future. In addition, this coding concept can be readily scaled to other frequency, e.g., the terahertz or optical range, offering simple but powerful approach for controlling the reflection and scattering of EM wave.

## Methods

### Simulations of the geometric phase coded metasurface

All the full-wave simulation results are analyzed with commercial software, CST Microwave Studio. In the meta-particle simulation, the unit cell is applied with periodic boundary condition along both *x*- and *y*-directions while open for *z*-direction in the free space. The electric field is applied either parallel to *x*- or *y*-direction to correspondingly obtain the meta-particle performances under the illumination of linearly *x*- or *y*- polarized waves, respectively.

### Experimental setup

The reflection measurements are carried out in a microwave anechoic chamber to minimize the reflections from surroundings as well as to avoid the interference from the environment. To obtain the backward scattering reduction attributed to the digital metasurface with randomized coding sequence, the scattering coefficient from the sample is calibrated to a bare same-sized metallic slab. A pair of horn antenna serving as the emitter and the detector is separated by microwave absorbing materials to get rid of near-field effect as well as the direct transmission between them. For normal incidence, the two antennas are placed adjacently at the angle of ±5° above the samples with a distance of 2 m to meet the condition of far-field measurements. Then, they are connected to the two ports of a vector network analyzer (Agilent N5245A) by coaxial-cables, respectively. By rotating with an angle of 90°, the horn antennas can be reconfigured to produce the normal illumination of linearly *x*- or *y*-polarized waves. To map the scattering pattern of the sample, the emitter is fixed at the angle of −5° while the receiver is moving along a circumference trace from −65° to 65° with a step of 10° to detect the EM wave scattered to different directions.

## Additional Information

**How to cite this article**: Chen, K. *et al.* Geometric phase coded metasurface: from polarization dependent directive electromagnetic wave scattering to diffusion-like scattering. *Sci. Rep.* **6**, 35968; doi: 10.1038/srep35968 (2016).
